# Contribution of SARS-CoV-2 infection preceding COVID-19 mRNA vaccination to generation of cellular and humoral immune responses in children

**DOI:** 10.3389/fimmu.2023.1327875

**Published:** 2023-12-20

**Authors:** Marije K. Verheul, Martijn Vos, Lia de Rond, Mary-Lène De Zeeuw-Brouwer, Kim H. Nijhof, Debbie Smit, Debbie Oomen, Petra Molenaar, Marjan Bogaard, Rianne van Bergen, Irene Middelhof, Lisa Beckers, Alienke J. Wijmenga-Monsuur, Anne-Marie Buisman, Mardi C. Boer, Rob van Binnendijk, Jelle de Wit, Teun Guichelaar

**Affiliations:** Centre for Infectious Disease Control, National Institute for Public Health and the Environment (RIVM), Bilthoven, Netherlands

**Keywords:** COVID-19 vaccine, children, adaptive, T cells, B cells, antibodies, SARS-CoV-2, infection

## Abstract

Primary COVID-19 vaccination for children, 5-17 years of age, was offered in the Netherlands at a time when a substantial part of this population had already experienced a SARS-CoV-2 infection. While vaccination has been shown effective, underlying immune responses have not been extensively studied. We studied immune responsiveness to one and/or two doses of primary BNT162b2 mRNA vaccination and compared the humoral and cellular immune response in children with and without a preceding infection. Antibodies targeting the original SARS-CoV-2 Spike or Omicron Spike were measured by multiplex immunoassay. B-cell and T-cell responses were investigated using enzyme-linked immunosorbent spot (ELISpot) assays. The activation of CD4^+^ and CD8^+^ T cells was studied by flowcytometry. Primary vaccination induced both a humoral and cellular adaptive response in naive children. These responses were stronger in those with a history of infection prior to vaccination. A second vaccine dose did not further boost antibody levels in those who previously experienced an infection. Infection-induced responsiveness prior to vaccination was mainly detected in CD8^+^ T cells, while vaccine-induced T-cell responses were mostly by CD4^+^ T cells. Thus, SARS-CoV-2 infection prior to vaccination enhances adaptive cellular and humoral immune responses to primary COVID-19 vaccination in children. As most children are now expected to contract infection before the age of five, the impact of infection-induced immunity in children is of high relevance. Therefore, considering natural infection as a priming immunogen that enhances subsequent vaccine-responsiveness may help decision-making on the number and timing of vaccine doses.

## Introduction

SARS-CoV-2 infection in healthy children is often asymptomatic or only accompanied by mild symptoms such as coughing or fever (1). However, SARS-CoV-2 infection in children can result in severe respiratory disease and other life-threatening diseases (2), such as multisystem inflammatory syndrome (MIS-C) (3, 4). mRNA vaccines are widely used to prevent COVID-19 and were among the first highly successful vaccines during the rise of the COVID-19 pandemic (5). Their efficacy against infection in children was reported to be 90% or higher (6), showing that SARS-CoV-2 vaccines can offer significant protection in these young age groups.

Timelines and policies with regards to the rollout of SARS-CoV-2 vaccines for children differed between countries (7). In the Netherlands, children 12-17 years of age without underlying health conditions were invited to receive a primary vaccination series against COVID-19 (two doses with the BNT162b2 mRNA vaccine, 30 µg each, advised interval 3 weeks) from July 2021. Subsequently, children 5-11 years of age without underlying health conditions were also invited for a primary series of vaccination (two doses with the BNT162b2 mRNA vaccine, 10 µg each, advised interval 4-8 weeks) from January 2022. Starting in the first months of 2022, the more infectious Omicron variant of concern (VOC) became the dominant variant in the Netherlands. This was paralleled by rapidly increasing numbers of reported cases of COVID-19 among all age groups (8). Therefore, a substantial number of children was expected to have had contracted an infection with one of the earlier VOC or the Omicron BA.1 variant before vaccination was offered.

In adults, it was shown that a previous SARS-CoV-2 infection combined with COVID-19 vaccination results in a much more elevated cellular and humoral immune response compared to vaccination alone (9–11). Compared to adults, children were reported to have similar or higher antibody levels in response to infection (12). Reports on cellular immune responses induced by SARS-CoV-2 infection in children and adults indicate the presence of either a suboptimal or comparable response in the younger population, potentially due to a milder disease course in the younger group (13–15). However, it remains questionable to what extent an infection preceding vaccination will impact cellular and humoral immune responses in children.

Given that a considerable proportion of children will have been infected prior to vaccination, it is important to better understand how the cellular and humoral immune responses to vaccination of younger persons are affected by infection prior to vaccination. These children are expected to have gained a degree of immunity due to encountering the virus prior to vaccination. Therefore, it is important to also investigate whether the administration of a second vaccine dose after the first dose will still result in enhancement of the immune response. For future use of mRNA immunizations in childhood vaccination programs, it is important to understand the immune response that is induced by such vaccines in young individuals after having contracted SARS-CoV-2 infection. Therefore, we studied the impact of prior SARS-CoV-2 infection on the cellular and humoral immune response generated by vaccination in children. The findings will provide additional insight into the size of the adaptive immune response induced by vaccination that may inform future vaccination strategies in the control of COVID-19.

## Materials and methods

### Study population

The participants studied were part of a larger prospective observational SARS-CoV-2 vaccination cohort recruiting participants up to 60 years of age by approaching people from the general population via several Personal Records Database (BRP) drawings (IIVAC study; NL76440.041.21, EudraCT: 2021-001357-31) (12). In addition, children 5-11 years of age were solicited to participate in the IIVAC study through recruitment via the Long COVID KIDS study (16) and were largely healthy controls from that study invited via BRP drawings and social media campaigns. Before inclusion, children were asked whether they experienced post-COVID-19 complaints. Of note, no such COVID complaints were reported by 5–11-year-old children before inclusion in this study. Children with cancer, transplants, chronic kidney disease, Down syndrome, HIV, auto immune diseases and/or any immune deficiency through disease or medication were excluded from participation in the study. Participant characteristics can be found in **Table 1**.

**Table 1:** Characteristics of the study population.

	All children	Infected before vaccination	Not infected before vaccination
**N**	64	30	34
**Age (mean(sd))**	12 (3)	10 (3)	13 (2)
**Sex (N female (%female))**	39 (61)	22 (73)	17 (50)
**1 vaccine / 2 vaccines (N)**	18/28	18/12	0/34
**Vaccine Interval (median days (range))**	28 (21-81)	56 (21 - 81)	28 (21-60)
**Paired samples before first vaccination and after first vaccination available (N)**	44	30	14
**Paired samples before second vaccination and after second vaccination available (N)**	43	9	34

sd, standard deviation.

Children received 10 µg/dose (5-11 years) or 30 µg/dose (12-17 years) of BNT162b2 (Comirnaty, Pfizer) by Public Health Services (GGD). Children were offered two vaccine doses, but some elected to only receive one vaccine dose. Blood samples (via finger prick or venipuncture) and questionnaires were taken prior to COVID-19 vaccination and at fixed intervals after vaccination. Questionnaires included demographic and SARS-CoV-2 testing information. Participants who reported a positive SARS-CoV-2 test within 28 days after receiving a vaccine and those who showed evidence of a new infection in this period based on the presence of nucleoprotein (NP) antibodies were excluded from the current analysis. In addition, participants were only included in the antibody analysis if at least two consecutive samples (one before and one after the first or the second vaccine dose) were available. For cellular assays, sample selection was limited by availability of PBMC samples. Within this availability, PBMC samples were selected to include participants with or without a prior infection, participants who received one or two vaccine doses and, if possible, those within each of these categories from both age groups (aged 5-11 and 12-17 years). We aimed for equal distribution of samples among these categories.

### Sample acquisition and processing

Serum and PBMC samples were collected before the first vaccination and 28 days after the first (± 2 days) and/or second (- 7 days till + 21 days) vaccination of the primary vaccination series. Serum samples were either self-collected by finger prick in microtubes and returned by mail, as described previously (17), or via venipuncture by a research nurse during a home visit. Once received by the laboratory at the National Institute for Public Health and the Environment (RIVM), the Netherlands, serum from microtubes and from blood sample tubes were spun down and frozen at -80°C as previously described (12). Heparin tubes were used to collect PBMCs (subgroup only), which were isolated with Lymphoprep as described previously (18).

### SARS-CoV-2 multiplex immunoassay

SARS-CoV-2 multiplex immunoassays were carried out to determine serum antibody levels towards SARS-CoV-2 Spike S1 (Sino Biological, 40591-V08H) and Spike S1 Omicron B1.1.529 (Sino Biological, 40591_V08H41) as described previously (19). In short, the serum was incubated with antigen-coupled beads for 45 minutes in the dark, followed by a 30-minute incubation with Goat-anti-human IgG. Samples were incubated in SM01 (Surmodics) and washing steps after each incubation were carried out with PBS. Antibody binding to antigen-coupled beads was determined with an FM3D (Luminex) and antibody levels were expressed as binding antibody units (BAU)/ml for S1 and as arbitrary units (AU)/ml for S1 Omicron BA.1. Antibody data for the 12–17-year-old participants in this study were published previously (12). The threshold for seropositivity was set at 10.1 BAU/mL for Spike S1 Wuhan and 14.3 BAU/mL for Nucleoprotein, as previously standardized for the Wuhan (vaccine) strain against the NIBSC/WHO COVID-19 reference serum 20/136. No cut-off has been established for Spike S1 Omicron antibodies.

### Interferon gamma T cell ELISpot

Interferon gamma (IFN-γ) ELISpot was adapted from a protocol described earlier (20). In brief, ethanol-activated Multiscreen HTS IP filter plates (Millipore; MSIPS4510) were coated with 5 μg/ml anti-human IFN-γ monoclonal antibodies (1-D1K, MabTech, 3420-3-1000) in PBS, overnight at 4°C. After washing with PBS, the plates were blocked using AIM-V medium (Gibco BRL, 12055-083) supplemented with 2% heat-inactivated human serum (Sigma, H6914) (AIM-V + 2%HS) for 1 hour. Then, PBMCs that were thawed using RPMI medium (Gibco, 52400-025) supplemented with 10% Fetal Bovine Serum (FBS; GE Life Sciences, SH30071.03) and 1% Penicillin/Streptomycin (Lonza, DE17-602E), were plated at 2x10^5^ cells/well (or 5x10^4^ cells/well for the positive control) in AIM-V + 2%HS. Cells were stimulated in duplicate or triplicate with peptide pool containing 15-mer overlapping peptides (11 amino acids overlap) spanning the entire Spike protein from the Wuhan strain or VOCs or the nucleoprotein from the Wuhan strain of SARS-CoV-2 (0.65 µg/ml; JPT), DMSO (negative control), or PHA (positive control; 1 µg/ml), for 22-24 hours, at 37°C, 5% CO_2_. Next, plates were washed with PBS + 0.05% Tween-20 and incubated with 1 μg/ml anti-human IFN-γ biotinylated detection antibody (7-B6–1, MabTech, 3420-6-1000) in PBS + 0.5% FBS for 1 hour. After washing with PBS + 0.05% Tween20, ELISpot plates were incubated with Extravidin Alkaline Phosphatase (1 μg/ml; Sigma, E2636) in PBS+0.5% FBS for 1 hour. Next, the plates were washed with PBS +0.05% Tween20, then rinsed with PBS, and developed with 5-bromo-4-chloro-3-indolyl phosphate (BCIP)/nitro blue tetrazolium (NBT) (SigmaFast; Sigma, B5655). The reaction was stopped after approximately 7 minutes by H_2_0 and the plates were dried in the dark. Spots were analyzed with an Immunospot C.T.L. reader and software. The number of spot-forming units (SFU) from DMSO controls was subtracted from total SFU counted in cultures with antigen. Samples that showed low response to PHA (< 33 SFU/5x10^4^ PBMC) compared to other samples from the same donor were excluded from the analysis due to technical issues. Finally, counted spots are represented as IFN-γ spot-forming units (SFU).

### SARS-CoV-2 B cell ELISpot

SARS-CoV-2 B cell ELISpots were carried out as described previously (18). In short, PBMC samples were cultured for 5 days in AIM-V medium with 10% heat-inactivated FBS, 50uM β mercaptoethanol, 3µg/ml CpG, 10ng/ml recombinant Interleukin 2 and 10ng/ml recombinant interleukin 10. ELISpot filtration plates (Millipore, MSIPS4510) were pre-wetted with 70% ethanol and coated with 10ug/ml of SARS-CoV-2 S1 (Sino biological, 40591-V08H), S1 Omicron BA.1 (Sino biological), MERS S1 (Sino Biological, 40069-V08H), or goat anti-human IgG (MP biomedicals, 855071). After overnight incubation of previously cultured cells on the pre-coated plates, antibody binding was detected with 0.2µm filtered 50 µl/well AP-conjugated goat-anti-human IgG (Seracare, 5220-0462), diluted in 1:5000 in PBS/0.01% Tween20/1% goat serum (ICN Biomedicals, 191356). Spots were made visible with BCIP (Sigma, B5655 or KPL, Seracare, 5420-0038) and subsequently imaged and counted using the same settings throughout each experiment (Immunospot C.T.L, Sample sensitivity = 185, Spot separation = 5, gating = 0.0133 to 0.3339, diffuse processing = large). No background subtraction was carried out and spot numbers were normalized to numbers of specific memory B-cells per 100.000 PBMCs.

### Flow cytometry

We analyzed the induction of activation markers OX40 (TNFRSF4, CD134) and CD154 (CD40L) by CD4^+^ T cells and activation markers OX40 (TNFRSF4, CD134) and CD137 (4-1BB) by CD8^+^ T cells in response to Spike peptides using flowcytometry. To this end, PBMCs that had been cultured with Spike peptides or with DMSO as unstimulated control for IFNγ ELISpot were collected after finishing the culture step of the ELISpot protocol. PBMCs were washed and stained using a cocktail containing a Live/dead dye (FVS780, BD Biosciences) and monoclonal antibodies to stain outer membrane markers using CD3-BUV496, CD4-BUV805, CD8-BUV395, OX40-BB700 (all BD Biosciences), CD154-PE-Cy7, and CD137-BV605 (both Biolegend) directly after collection of the cells. After subsequent washing, the cells were analyzed on a Symphony A3 flow cytometer (BD Biosciences). Data were analyzed with FlowJo software Version 10 (BD Biosciences) to determine frequencies of cells showing dual expression of activation markers among the live CD3^+^CD4^+^ lymphocytes and the live CD3^+^CD8^+^ lymphocytes (**Supplementary Figure S4**). Responsiveness to Spike antigen per participant was calculated by subtracting the frequency of positive cells in the DMSO control from the frequency of positive cells after culture with Spike antigen.

### Statistics

Data were analyzed in R (version 4.3.0) or Graphpad Prism (version 9.5.1). Appropriate tests to investigate statistical significance are described in the respective figure legends.

## Results

### Study population characteristics

Participants were invited to receive their first vaccine dose as part of the primary vaccination series from August 2021 through August 2022. Thirty out of 64 (47%) participants were classified as infected based on a self-reported positive COVID-19 test or the presence of antibodies in serum to Spike S1 or nucleoprotein before receiving the first vaccination. ELISpot data indicate that the sum of T-cell responsiveness to Spike + NP prior to vaccination confirms that children included in our cellular assays and classified as not infected based on antibodies and COVID-19 tests were naive to SARS-CoV-2 (**Supplementary Table S1**). In samples of all twelve children classified as not previously infected and of which ELISpot data were complete, the sum of spot counts to Spike + NP (0-7 SFU/2x10^5^ PBMC) was lower than this sum of spot counts in samples from all children classified as previously infected (9-55 SFU/2x10^5^ PBMC). SARS-CoV-2-variants of concern Delta (Summer 2021 to Winter 2021/2022) and Omicron (Winter 2021/2022 to Summer 2022 and beyond) were dominant in the Netherlands before and during the vaccination period. The majority of vaccinated participants 5-11 years of age for whom vaccination became available from January 2022 were classified as infected before vaccination (21 of 23 children; 91%). For participants 12-17 years of age, vaccinated from July 2021 onwards, 9 of 41 participants (22%) had been infected before vaccination. All uninfected participants received two vaccine doses, while 18 of the 30 infected participants (60%) received only one dose (**Table 1**).

### Vaccination induces antibody responses in both naive and previously infected children

**Figure 1:**
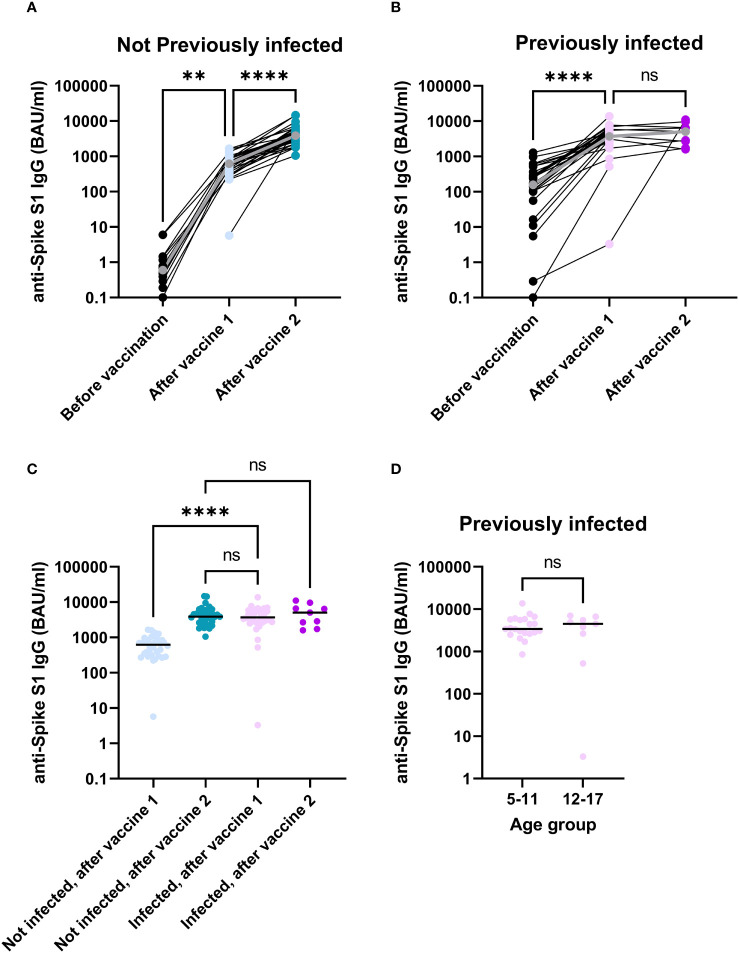
In children, SARS-CoV-2-specific antibodies significantly increase through immunization. Anti-Spike S1 antibodies detected in serum of participants without a previous infection **(A)** or with a previous infection **(B)**. A comparison between infected and non-infected participants after their first or second vaccine dose **(C)**. Antibody levels were compared between participants 5-11 years of age and participants 12-17 years of age in previously infected participants 28 days after their first vaccination **(D)**. The grey dots and lines for **(A)** and **(B)** represent median values. The horizontal lines for **(C, D)** indicate median values. Difference for panels **(A–C)** were investigated with a Kruskal-Wallis test with Dunn’s correction for multiple comparisons. Groups in panel **(D)** were compared with a Man-Whitney U test. ns, not significant; **, p < 0.01; ****, p < 0.0001; BAU, Binding antibody units.

To investigate to what extent vaccination induces antibody responses to SARS-CoV-2, levels of Spike S1-specific antibodies were determined before vaccination, after the first vaccine dose and after the second vaccine dose. Participants without a previous infection showed robust antibody responses after each vaccine dose (**Figure 1A**, p = 0.003 for the first dose, p < 0.0001 for the second dose) with median antibody levels increasing from 0.6 BAU/ml (n = 14) at baseline, to 618.4 BAU/ml (n = 34) after the first vaccine dose and 3853.2 BAU/ml (n = 34) after the second vaccine dose. Participants who had been infected prior to vaccination also showed an increased antibody response after the first vaccine dose (**Figure 1B**, p < 0.0001), with antibody levels increasing from 156.8 BAU/ml (n = 30) to 3699.305 BAU/ml (n = 30), indicating that vaccination of these previously infected children further enhances specific antibody responses to the Spike S1 antigen. Antibody levels, however, did not significantly increase further after a second vaccine dose (**Figure 1B**, p = 1, 5058.6 BAU/ml, n = 9). Notably, antibody levels after one vaccine dose were higher for participants who were previously infected compared to those who were not (**Figure 1C**, p < 0.0001). Furthermore, after one dose, previously infected participants already had antibody levels comparable to uninfected participants who received two vaccine doses (**Figure 1C**, p = 1), indicating that a second vaccine dose as part of the primary immunization series does not contribute significantly to the antibody response in previously infected participants. Within the infected group, no differences were observed between age groups after one vaccine dose (**Figure 1**, p = 1). Nevertheless, the first vaccine dose induces robust levels of SARS-CoV-2-specific antibodies in the current population, and these levels are higher in children who previously experienced an infection compared to naive children.

### Both infection and vaccination contribute to memory B cell responses in children

**Figure 2:**
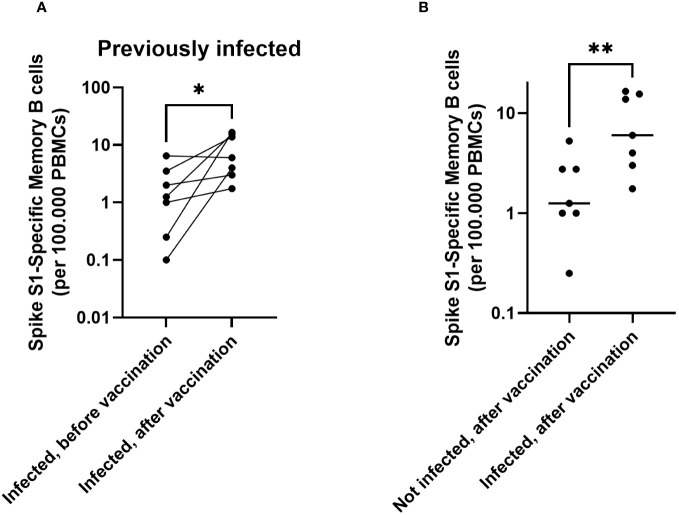
In children, both infection and immunization contribute to memory B cell responses towards SARS-CoV-2. Spike S1-specific memory B cells are shown for participants with a previous infection (n = 7), before vaccination and 28 days after vaccination (pairwise) **(A)**. Comparison of memory B cells after vaccination for those with (n = 7, right) and without an infection history (n = 7, left) **(B)**. The horizontal lines for panel **(B)** indicate the medians. For plotting purposes, zeros were assigned to 0.1. Differences between paired samples were investigated using a Wilcoxon Signed rank test. Differences between unpaired groups were investigated with a Mann Whitney U test. * = p < 0.05, ** = p < 0.01.

As memory B cells are important for antibody production upon repeated antigen contact, SARS-CoV-2 Spike-specific memory B cells were quantified by ELISpot in a subset of participants to detect their presence and their dynamics in response to vaccination. Memory B-cell data are available from a subset of participants with a previous infection before and after vaccination (n = 7). For participants without a previous infection, memory B cell data are available after vaccination only (n=7) (**Supplementary Table S2**). All previously infected participants showed an increase in memory B-cell responses after vaccination (**Figure 2A**, p = 0.03). In a direct comparison to participants who were not previously infected, a previous infection resulted in higher numbers of Spike S1-specific memory B cells after vaccination (**Figure 2B**, p = 0.009. No differences were noted in memory B cell responses between different age groups or between participants who received one or two vaccine doses (**Supplementary Figures S1A**, **S2A**). These data show a limited induction or maintenance of memory B cells in peripheral blood upon infection. However, upon vaccination, significant boosting of spike specific memory B cells was observed.

### Vaccination induces T-cell responses in both naive and previously infected children

**Figure 3:**
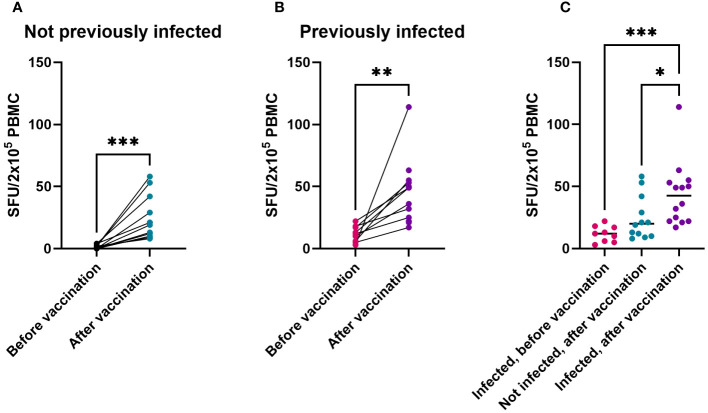
T-cell response to Spike from Original Vaccine Strain is induced in both naive and previously infected children, as shown by IFN-γ ELISpot. IFN-γ-producing cells among PBMC cultured in the presence of overlapping peptides of the Spike antigen from SARS-CoV-2 Original Strain, expressed as spot forming units (SFU) corrected for DMSO controls. SFU before vaccination and after vaccination in children without SARS-CoV-2 infection prior to vaccination **(A)** or in children with SARS-CoV-2 infection prior to vaccination **(B)**. Individual SFU (dots) and their median values in PBMC from children infected prior to their vaccination (‘Infected’), from uninfected children after their vaccination, or from previously infected children after their vaccination **(C)**. Statistical significance of differences was analyzed by Wilcoxon test **(A, B)** and Kruskall-Wallis test with Dunn’s correction for multiple testing **(C)**. * = p < 0.05, ** = p < 0.01, *** = p < 0.001.

Spike-specific T-cell numbers measured by ELISpot increased significantly after vaccinations, both in not previously infected (**Supplementary Table S3**, **Figure 3A**, p = 0.001) and in previously infected children (**Figure 3B** (p = 0.004). Compared to non-infected vaccinees, exposure to SARS-CoV-2 before vaccination resulted in a higher T-cell response to primary vaccination (**Figure 3C**, p = 0.048). Differences between the two age groups (**Supplementary Figure S1B**) or between previously infected children who received one or two vaccine doses (**Supplementary Figure S2B**) were not statistically significant. Altogether, these data show that vaccination significantly contributes to Spike-specific T-cell responses in children, and that these T-cell responses become even stronger in those who had been infected with SARS-CoV-2 prior to vaccination.

### Infection and vaccination induce distinct T-cell responses in the CD4^+^ and CD8^+^ populations

**Figure 4:**
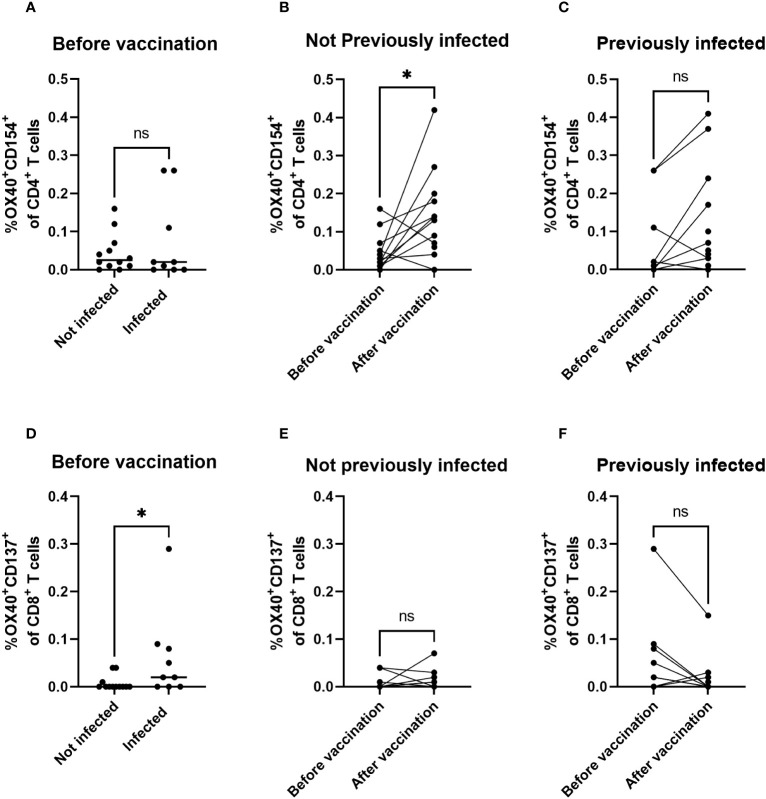
Infection skews towards CD8^+^ T-cell responsiveness, whereas vaccination skews to CD4^+^ T-cell responsiveness. Response of CD4^+^ T cells and CD8^+^ T cells to Spike antigen from SARS-CoV-2 Original Strain as measured by Spike-peptide-mediated induction of dual expression of activation markers OX40 and CD154 among CD4^+^ T cells **(A–C)** or OX40 and CD137 among CD8^+^ T cells **(D–F)** using flow cytometry. Net percentages per individual participant are shown by dots (and calculated from percentage positive cells in presence of Spike antigen minus percentage of positive cells in DMSO control) and horizontal bars show their median values per group indicated. Statistical significance of differences was analyzed by Wilcoxon **(B, C, E, F)** or Mann-Whitney **(A, D)**. ns, not significant; *, p < 0.05.

To gain insight in the type of T cells that respond to vaccination, we analyzed the induction of activation markers expressed by CD4^+^ and CD8^+^ T cells in response to Spike peptides by flow cytometry. Naive and SARS-CoV-2-infected children showed comparable frequencies of responding CD4^+^ T cells prior to vaccination (**Figure 4A**, p = 0.8). In naive children, frequencies of responding CD4^+^ T cells were significantly elevated after primary vaccination (**Figure 4B**, p = 0.02), whereas in previously SARS-CoV-2-infected children a slight but unsignificant elevation of the frequency of responding CD4^+^ T cells was observed after vaccination (**Figure 4C**, p = 0.08). Remarkably, previous infection with SARS-CoV-2 induced significantly higher frequencies of responding CD8^+^ T cells prior to vaccination as compared to naive participants (**Figure 4D**, p = 0.03). In contrast to CD4^+^ T cells, no significant vaccine-induced changes of responsive CD8^+^ T cells were detected, both in non-infected (**Figure 4E**, p = 0.7) and previously infected children (**Figure 4F**, p = 0.1). Together, these data imply that primary mRNA COVID-19 vaccination may drive T-cell responsiveness towards the CD4^+^ subset, whereas a priming SARS-CoV-2 infection may drive an increase in T-cell responsiveness more profoundly in the CD8^+^ subset.

### Original Spike antigen generates responsiveness to (Spike protein of) Omicron variant of concern

**Figure 5:**
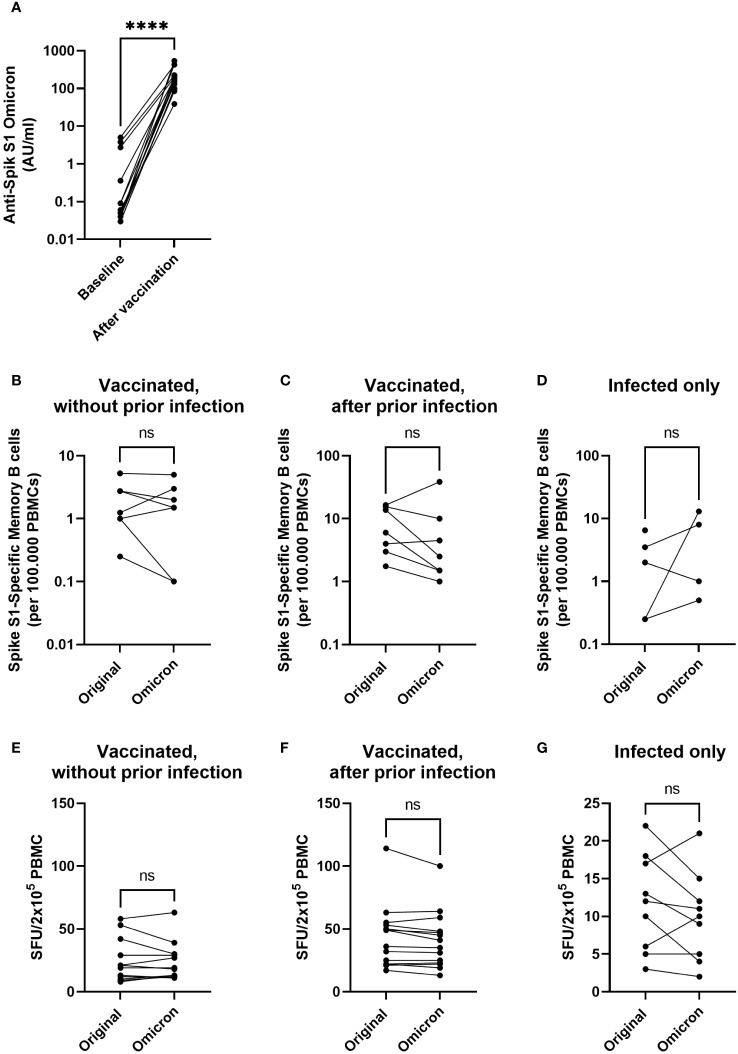
Vaccination with Original Strain antigen induces an omicron-specific antibody response and comparable B- and T-cell responses to the original and Omicron Spike protein. Anti-SARS-CoV-2 Omicron B1.1.529 antibodies were measured before and after completion of the primary vaccination series in participants without evidence of a previous infection (n = 12) **(A)**. Specific memory B cells targeting the original Spike S1 or Omicron BA.1 Spike S1 were investigated using B cell ELISpot for participants who were vaccinated without prior infection **(B)**, vaccinated after a prior infection **(C)**, or only infected without a vaccination **(D)**. IFN-γ-producing cells among PBMCs cultured in the presence of overlapping peptides of the Spike antigen from SARS-CoV-2 Original Strain or Omicron are expressed as spot forming units (SFU) **(E–G)**. Graphs show SFU of IFNγ spots per 2x10^5^ PBMC after vaccination in participants without **(E)** or with SARS-CoV-2 infection **(F)** prior to vaccination, or in previously infected children before their first vaccination **(G)**. Statistical significance of differences was analyzed by a paired Wilcoxon test. ns, not significant; ****, p < 0.0001.

Finally, we assessed whether T-cell and B-cell responses of children generated by the original strain Spike antigen from the BNT162b2 mRNA vaccine would also engraft recognition of Spike proteins from later SARS-CoV-2 variants of concern. Of all dominant strains circulating at the time of sample collection, Omicron was antigenically most diverted from the Original Strain in the evolution of SARS-CoV-2. Vaccination with the original Spike antigen resulted in an increase in antibodies targeting the Spike S1 from Omicron B1.1.529 (**Figure 5A**, p < 0.001). Furthermore, no significant differences were observed in vaccine-induced responses between the original and the Omicron variant of Spike for both B cells (**Figures 5B–D**) and T cells (**Figures 5E–G**), indicating a substantial cross-reactive cellular response against the Omicron variant is induced in children by vaccination with BNT162b2. Moreover, in both naive and previously SARS-CoV-2-exposed children, vaccine-induced T-cell responses to original strain Spike strongly correlated with T-cell responses observed to Omicron (**Supplementary Figure S3**). These findings suggest that T cells addressed by Original Strain Spike antigen are likely to respond to a large extent to currently occurring new variants of concern. Combined, these observations indicate that primary vaccination with Original Spike antigen generates similar cellular response to Spike protein of the more distant Omicron variant of concern in children.

## Discussion

In this study, we characterized cellular and humoral immune responses to COVID-19 mRNA vaccination and show that the BNT162b2 mRNA vaccine induces antibody, B-cell, and T-cell responses to Spike antigen in both naive and previously infected children (5-17 years of age). Moreover, children who had contracted SARS-CoV-2 infection prior to vaccination showed even higher responsiveness of T cells and B cells, and required less vaccine doses to reach a peak antibody response. In addition, the response induced by the vaccine containing the original SARS-CoV-2 Spike antigen was cross-reactive to Spike of variant of concern Omicron, as we observed an increase in antibody levels after vaccination and a strong correlation of the overall T-cell responses to both Spike variants.

Our study confirms that in infection-naive children a second vaccine dose will further increase antibody levels. In contrast, no significant increase in antibody levels was induced by the second vaccine dose in previously infected children. This observation contrasts with studies among the general population, where the second vaccine dose in previously infected participants over 18 years of age induces a further increase of IgG levels that can even exceed concentrations induced by two doses in infection-naive participants (12, 17, 21). These studies assessed antibodies in cohorts that were of a broader and older age range, indicating that our findings may be confined to a younger age group. Thus, infection prior to vaccination has a significant impact on antibody levels induced by COVID-19 mRNA vaccination in children. However, Cinicola et al. investigating the role of infection in 5–11-year-old children did observe that previously infected children had increased antibody levels 1 to 2 weeks after receiving a second vaccination (22). We did not find evidence for difference in antibody levels between groups after the second vaccination, but this may be explained by differences in the timing of the measurements after vaccination, different intervals between vaccine doses, or alternate criteria to classify participants as infected. Although cellular responses in our study tended to be slightly higher in previously infected children who received two doses compared to those who received one dose, we observed no significant difference between cellular responsiveness to one and two doses. The number of participants in our cohort may have been too low to detect subtle increases in response to the second dose in previously infected children. In addition, differences may exist between age groups, as the youngest age group (5-11 years) received a dose of 10 µg, while the older age group received 30 µg. Therefore, larger cohorts are needed to draw firm conclusions on cellular responses to one versus two doses in children with a history of SARS-CoV-2 infection.

Further comparisons of data from our study to previous findings for adults may provide additional insight in how children and adults may respond to vaccines differently. Part of the antibody data measured for 12–17-year-old participants has previously been published in a study showing that the antibody levels in younger individuals are generally higher, compared to those in (older) adults (12). Interestingly, the numbers of memory B cells in children are lower compared to those we previously determined in adults (18-50 years of age) after two vaccine doses without a previous infection (18) (median = 1.25/1*10^5^ PBMC for children (n = 7) and 10.5/1*10^5^ PBMC for adults (n = 32). This potentially indicates that B cells in children are more likely to become antibody-producing plasmablasts after COVID-19 mRNA vaccination, while B cells in adults may be more likely to differentiate into memory B cells. Additional research into the development and dynamics of specific B and T cell subsets would be of interest to further explore this hypothesis.

T-cell responses to SARS-CoV-2 have been reported to be important to resolve SARS-CoV-2 infection and to control severity of disease (23–25). Responses of CD4^+^ and CD8^+^ T cells are known to be induced by mRNA vaccines and/or SARS-CoV-2 infection in adults (26–31). By using additional flow cytometry in our study, we found spike-responsive CD4^+^ T cell frequencies to increase mainly after vaccination and spike-responsive CD8^+^ T cell frequencies to increase by infection. This finding indicates skewing of responsiveness by vaccine versus infection in children. To our knowledge, such infection and vaccination-dependent skewing has not been reported for children before. We may have not detected all responding T cells due to alternate sensitivity of our assay compared to other types of flowcytometry assays and possible interference of IFN-γ-consumption herein due to the preceding ELISpot. However, it has been suggested that some vaccine platforms may induce CD8^+^ T-cell responses at lower levels than CD4^+^ T-cell responses (28). This tendency can also be observed under some assay conditions, specifically for mRNA vaccines (29). Moreover, the skewing may be explained by different responsiveness at young age compared to (older) adults that have been subject of most studies today. This was exemplified by a recent study among age range 18-82 years in which it was found that the frequency of infection-induced CD4^+^ T cells significantly drop with lower age (15). Moreover, compared to adults, children have been reported to express higher frequencies of naive CD8^+^ T cells and better antigen-presentation via innate cells. In addition to mere age-related immune development, skewing towards CD8^+^ T cells to infection at younger age can also be explained by the course of disease during COVID-19 generally being milder in children. Namely, studies in adults have shown that milder cases of COVID-19 correlate with lower CD4^+^ T-cell responsiveness (30, 31) and higher CD8^+^ T-cell responsiveness (31). Together, these findings suggest that the T-cell subsets of children respond different than those of adults, and may exert stronger CD8^+^ T-cell responses upon infection to clear virus-infected cells.

Although new virus variants of concern such as Omicron may partly evade immune reactivity of antibodies and T cells generated by original strain Spike antigen in the vaccine (25, 32–34), some studies reported that such immune evasion may not largely contribute to the overall T-cell response to spike antigen (29). We observed significant cross-reactivity of vaccine-induced immune responses to VOC Omicron in children as indicated by significant antibody response and strong correlation of T-cell response to Omicron Spike. However, one of the limitations of the current study is that we did not look at neutralization, which is expected to be lower (but not completely absent) for the omicron variant (32, 35). Nevertheless, it is likely that the SARS-CoV-2 mRNA vaccines containing the original strain may still provide some protection in children against currently circulating variants. Indeed, studies in children between 5 and 12 years of age confirm that vaccine efficacy against symptomatic infection with the omicron variant is significantly reduced compared to the delta variant (36, 37). Furthermore, there are indications that vaccine efficacy against the Omicron variant in children may be short-lived (36). It would therefore be relevant to further investigate SARS-CoV-2 vaccine-dependent humoral and cellular immune responses in children, although this will be complicated by infection rates and newly emerging variants.

Combined, our data show that infection can be considered as a priming immunogen capable of inducing an adaptive immune response that can be boosted by vaccination. This concept has recently been shown in adults (10, 11), and our study shows that this is applicable at a younger age. Currently, most children will likely have encountered SARS-CoV-2 infection before the age of five years as the virus is highly circulating endemically. Since infection has a significant stimulatory impact on responsiveness to subsequent vaccination, this immunological prime-boost concept by infection-vaccination is valuable for considerations on redefining vaccination programs for children.

## Data availability statement

The original contributions presented in the study are included in the article/**Supplementary Material**. Further inquiries can be directed to the corresponding author.

## Ethics statement

The studies involving humans were approved by Medical Research Ethics Committee Utrecht. The studies were conducted in accordance with the local legislation and institutional requirements. Written informed consent for participation in this study was provided by the participants’ legal guardians/next of kin.

## Author contributions

MKV: Conceptualization, Formal analysis, Investigation, Supervision, Validation, Visualization, Writing – original draft, Writing – review & editing, Data curation. MV: Formal analysis, Investigation, Writing – review & editing, Data curation, Methodology. LdR: Data curation, Formal analysis, Investigation, Methodology, Writing – review & editing. M-LD: Data curation, Formal analysis, Investigation, Methodology, Writing – review & editing. KN: Data curation, Formal analysis, Investigation, Methodology, Writing – review & editing. DS: Investigation, Methodology, Writing – review & editing, Project administration, Resources. DO: Investigation, Methodology, Project administration, Resources, Writing – review & editing, Formal analysis. PM: Formal analysis, Investigation, Methodology, Writing – review & editing, Data curation. MjB: Data curation, Formal analysis, Investigation, Methodology, Writing – review & editing. RiB: Methodology, Writing – review & editing. IM: Methodology, Writing – review & editing. LB: Writing – review & editing, Project administration, Resources. AW-M: Project administration, Resources, Writing – review & editing. A-MB: Writing – review & editing, Conceptualization, Investigation, Supervision. MCB: Conceptualization, Investigation, Supervision, Writing – review & editing, Funding acquisition, Project administration. RoB: Conceptualization, Investigation, Supervision, Writing – review & editing, Methodology. JdW: Conceptualization, Investigation, Supervision, Writing – review & editing, Funding acquisition. TG: Conceptualization, Investigation, Supervision, Writing – review & editing, Data curation, Formal analysis, Methodology, Validation, Visualization, Writing – original draft.

## Supplementary material

Supplementary Table 1 Classification of participants included in cellular assays​.

Supplementary Table 2 Characteristics of the study subpopulation selected for B cell assays.

Supplementary Table 3 Characteristics of the study subpopulation selected for T cell assays​.

Supplementary Figure 1 No differences in cellular response are detected between age groups. Spike S1-specific Memory B cells in participants with a previous infection, split between two age groups **(A)**. T-cell ELISpot showing response to Spike peptides among PBMC from previously infected children 28 days after receiving two shots of COVID-19 vaccine, split between two age groups **(B)**. The horizontal lines indicate the medians. For plotting purposes, zeros in the Memory B-cell ELISpot assay were assigned to 0.1. Differences between unpaired groups were investigated with a Mann Whitney U test. ns = not significant.

Supplementary Figure 2 Two immunizations after SARS-CoV-2 infection does not result in higher cellular responses. Spike S1-specific Memory B cells from previously infected children comparing 1 and 2 vaccine doses **(A)**. T-cell ELISpot response to Spike peptides among PBMC from previously infected children (age group 5-11 years) comparing one and two vaccine doses **(B)**. The horizontal line indicates the median. Differences between unpaired groups were investigated with a Mann Whitney U test. ns = not significant.

Supplementary Figure 3 Correlation of T-cell ELIspot response to Spike of Original Strain and Omicron variant of concern. Linear regression analysis of IFN-y-producing cells among PBMC cultured in the presence of overlapping peptides of the Spike protein from SARS-CoV-2 Original Strain and Omicron variant of concern B1629. Data are expressed as spot forming units (SFU) in PBMC samples of vaccinated children after vaccination.

Supplementary Figure 4 Flow cytometry gating. Definition of CD4^+^ T cells and CD8^+^ T cells among cultured PBMC analyzed by flow cytometry **(A)**. Gating of OX40^+^CD154^+^ among the CD4^+^ T cell subset **(B)** or OX40^+^CD137^+^ cells among the CD8^+^ T cell subset **(C)** after culture with SARS-CoV2-Spike peptides or DMSO of representative participants.
