# Supplementary material for: Contribution of SARS-CoV-2 infection preceding COVID-19 mRNA vaccination to generation of cellular and humoral immune responses in children
Source: Front Immunol. 2023 Dec 20;14:1327875. doi: 10.3389/fimmu.2023.1327875 (PMC10773747; doi:10.3389/fimmu.2023.1327875)
Supplement: Supplementary Table 1 — Classification of participants included in cellular assays​. [file DataSheet_1.pdf]

Supplementary tables and figures

Table S1. Classification of participants included in cellular assays

	Classified as *not infected* pre-vaccination												
**Child #**	**1**	**2**	**3**	**4**	**5**	**6**	**7**	**8**	**9**	**10**	**11**	**12**	**13**
Confirmed SARS-CoV2	No	No	No	No	No	No	No	No	No	No	No	No	No
Anti-S antibodies	No	No	No	No	No	No	No	No	No	No	No	No	No
Anti-NP antibodies	No	No	No	No	No	No	No	No	No	No	No	No	No
NP+S specific IFN-γ spots	1	0	5	0	0	0	0	5	0	7	3	2	?

	Classified as *infected* pre-vaccination													
**Child #**	**14**	**15**	**16**	**17**	**18**	**19**	**20**	**21**	**22**	**23**	**24**	**25**	**26**	**27**
Confirmed SARS-CoV2	Yes	Yes	Yes	Yes	Yes	Yes	Yes	Yes	No	No	No	No	No	No
Anti-S antibodies	Yes	Yes	Yes	Yes	Yes	No	?	No	Yes	Yes	Yes	Yes	Yes	No
Anti-NP antibodies	Yes	Yes	Yes	Yes	Yes	No	?	No	Yes	Yes	Yes	Yes	No	Yes
NP+S specific IFN-γ spots	23	55	?	?	?	9	?	23	12	13	?	39	9	11

Legend:
- No
- Yes
- ? Unknown

Table S2. Characteristics of the study subpopulation selected for B cell assays

	All children	Infected before vaccination	Not infected before vaccination
N	14	7	7
Age (mean(sd))	12 (3)	12 (3)	13 (4)
Sex (N female (% female))	8 (57)	4 (57)	4 (57)
1 vaccine / 2 vaccine (N)	3 // 11	3 // 4	0 // 7

Table S3. Characteristics of the study subpopulation selected for T cell assays

	Infected before vaccination		Not infected before vaccination	
	pre-vaccination	post-vaccination	pre-vaccination	post-vaccination
N	11	14	12	13
Median Age (sd)	12 (1) years	11 (3) years	13 (3) years	13 (3) years
Sex (N female (% female))	5 (45)	8 (57)	4 (33)	5 (38)
1 vaccine / 2 vaccines (N)	5 / 6	6 / 8	0 / 12	0 / 13

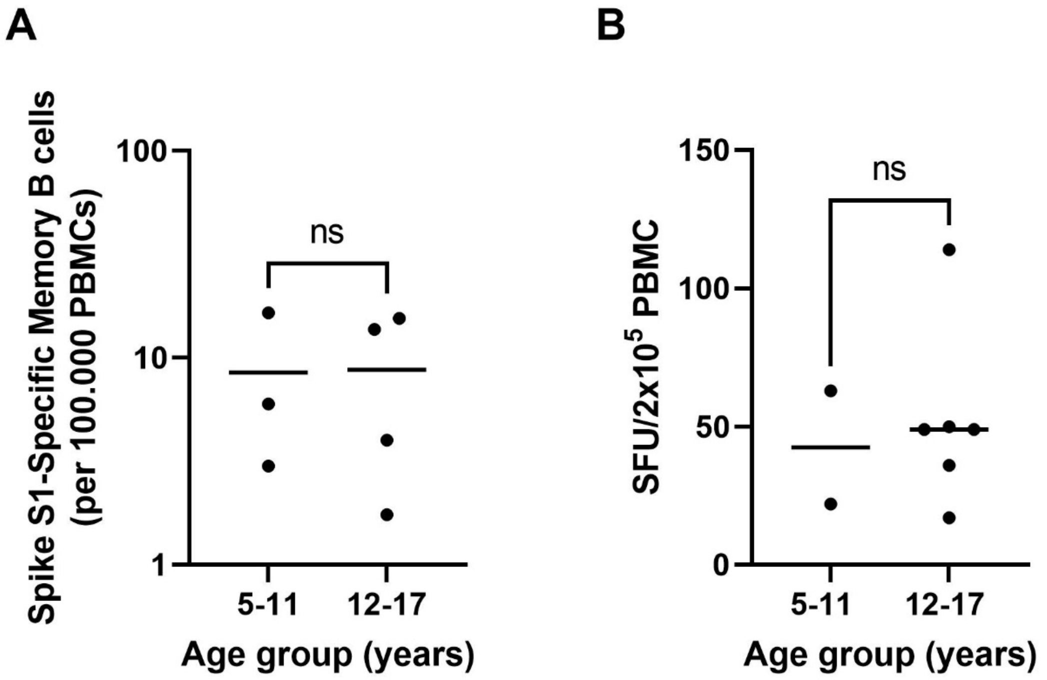

Figure S1. No differences in cellular response are detected between age groups.
Spike S1-specific Memory B cells in participants with a previous infection, split between two age groups (A). T-cell ELISpot showing response to Spike peptides among PBMC from previously infected children 28 days after receiving two shots of COVID-19 vaccine, split between two age groups (B). The horizontal lines indicate the medians. For plotting purposes, zeros in the Memory B-cell ELISpot assay were assigned to 0.1. Differences between unpaired groups were investigated with a Mann Whitney U test. ns = not significant.

Supplementary FIGURE S1

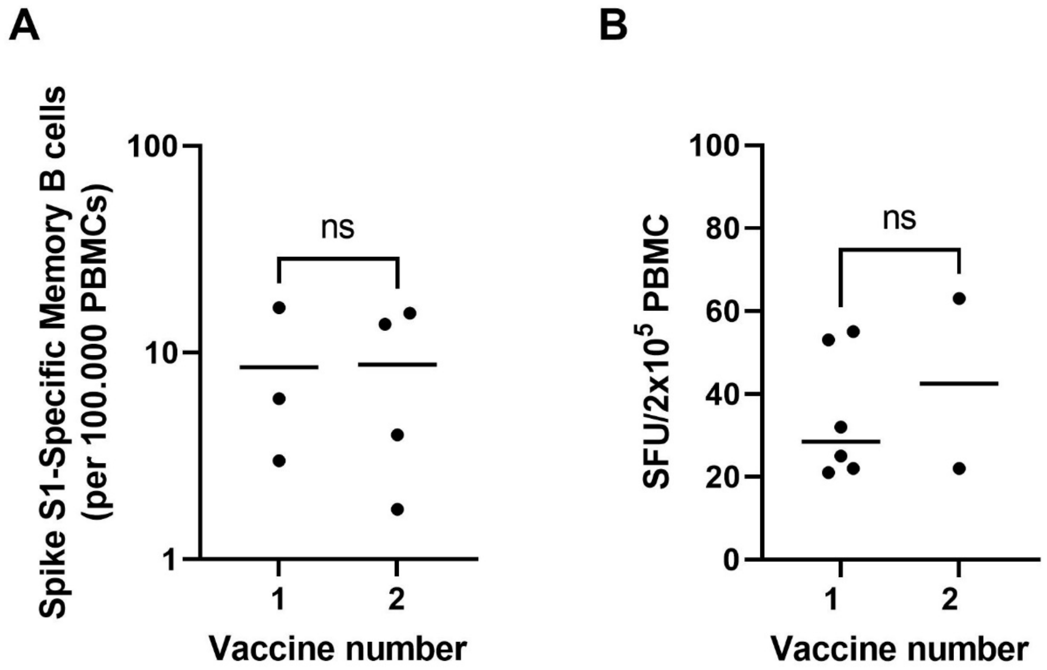

Figure S2. Two immunizations after SARS-CoV-2 infection does not result in higher cellular responses. Spike S1-specific Memory B cells from previously infected children comparing 1 and 2 vaccine doses (A). T-cell ELISpot response to Spike peptides among PBMC from previously infected children (age group 5-11 years) comparing one and two vaccine doses (B). The horizontal line indicates the median. Differences between unpaired groups were investigated with a Mann Whitney U test. ns = not significant

Supplementary FIGURE S2

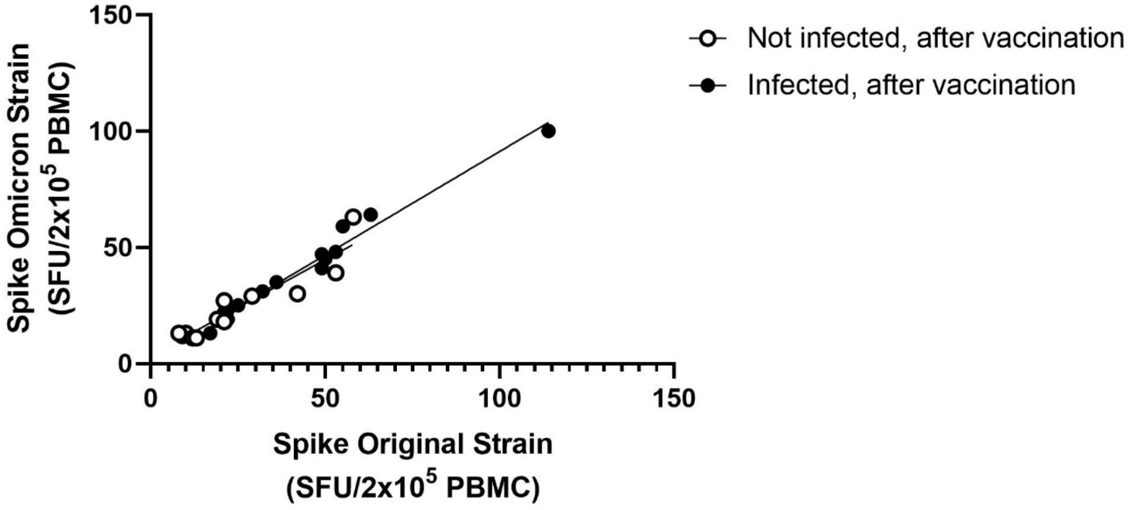

	Not infected	Infected
R squared	0.8671	0.9753

	Not infected	Infected
P value	<0.0001	<0.0001

Figure S3. Correlation of T-cell ELIspot response to Spike of Original Strain and Omicron variant of concern. Linear regression analysis of IFN-γ-producing cells among PBMC cultured in the presence of overlapping peptides of the Spike protein from SARS-CoV-2 Original Strain and Omicron variant of concern B1629. Data are expressed as spot forming units (SFU) in PBMC samples of vaccinated children after vaccination.

Supplementary FIGURE S3

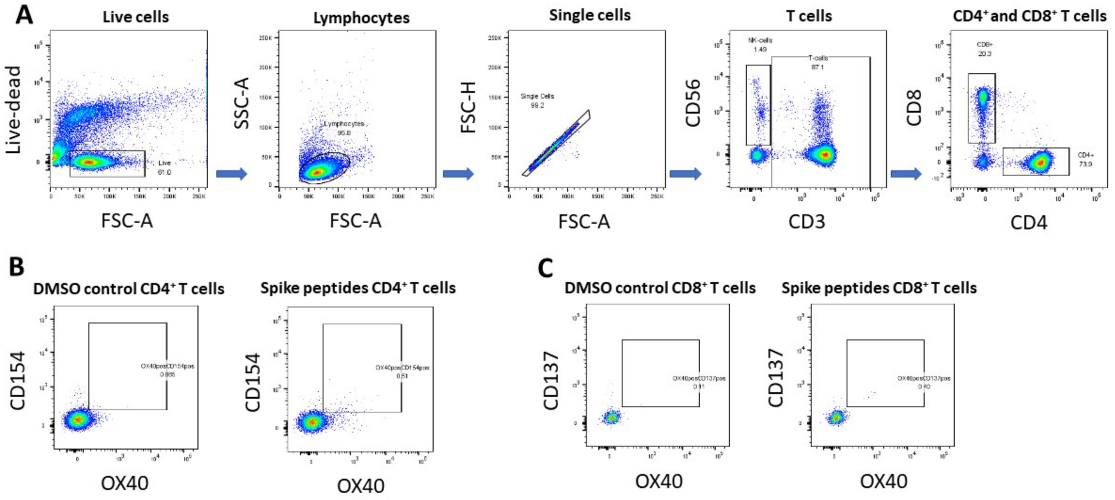

Figure S4. Flow cytometry gating.

Definition of $CD4^+$ T cells and $CD8^+$ T cells among cultured PBMC analyzed by flow cytometry (A). Gating of $OX40^+CD154^+$ among the $CD4^+$ T cell subset (B) or $OX40^+CD137^+$ cells among the $CD8^+$ T cell subset (C) after culture with SARS-CoV2-Spike peptides or DMSO of representative participants.

Supplementary FIGURE S4
